# Assessing the realism and face validity of Fix For Life: an embalmed human cadaver model for high-fidelity laparoscopic training

**DOI:** 10.1007/s00464-025-12550-2

**Published:** 2026-01-26

**Authors:** A. Masie Rahimi, Michael van Emden, Sem F. Hardon, Maarten Simons, H. Jaap Bonjer, Tim Horeman, Freek Daams

**Affiliations:** 1 Department of Surgery, Amsterdam UMC, Location Vrije Universiteit, Amsterdam, The Netherlands; 2 Science Hub for ASC Research and Education (SHARE), Amsterdam Skills Centre for Health Sciences (ASC), Amsterdam, The Netherlands; 3 Cancer Center Amsterdam, Amsterdam, The Netherlands; 4 Department of Anatomy and Neurosciences, Amsterdam UMC – VU University Medical Center, Amsterdam, The Netherlands; 5 Department of Surgery, Onze Lieve Vrouwe Gasthuis, Amsterdam, The Netherlands; 6 Department of Biomechanical Engineering, Delft University of Technology, Delft, The Netherlands; 7 Amsterdam UMC – Amsterdam Skills Centre, Tafelbergweg 47, 1105 BD Amsterdam, The Netherlands

**Keywords:** Laparoscopy training, Wet lab training, Cadaver training, Embalming method

## Abstract

**Background:**

Human cadaver simulation is vital in medical training, offering realistic experience crucial for skill development, especially in laparoscopic surgery. Traditional cadaver types, like fresh frozen and embalmed, have limitations. Fix4Life (F4L), a novel embalming technique, aims to overcome these drawbacks by providing flexible, pliable tissue without discoloration. This study evaluates the realism and face validity of the F4L embalmed cadaver model for laparoscopic training, aiming to enhance surgical education and patient safety.

**Methods:**

Surgical residents and expert surgeons from Amsterdam UMC participated in a hands-on laparoscopy course, performing laparoscopic appendectomy, cholecystectomy, and totally extraperitoneal (TEP) hernia repair on Fix4Life cadavers. Prior to this, residents completed questionnaires immediately after training, while experts reviewed procedure videos and provided evaluations. Ethical approval was obtained, and written consent was acquired from participants. Procedures were supervised, recorded, and securely shared for assessment. Face validation forms were filled by both novices and experts, assessing realism and key aspects of laparoscopic surgery. Statistical analysis included non-parametric tests due to non-normal data distribution.

**Results:**

Both residents and experts rated the laparoscopic procedures positively, with the TEP receiving particularly high scores. Residents rated the laparoscopic appendectomy and cholecystectomy as “Good” for all assessment points, while the TEP was frequently rated as “Very Good”. The experts also rated the procedures in the majority of cases as “Good”. Furthermore, novices tended to rate the procedures more favorably than experts, particularly in terms of lifelike tissue manipulation (*p* < 0.001), tissue color (*p* = 0.014), and comparability to reality (*p* = 0.046).

**Conclusion:**

The Fix4Life embalming method provides a realistic training modality for laparoscopic appendectomy, laparoscopic cholecystectomy, and TEP.

**Supplementary Information:**

The online version contains supplementary material available at 10.1007/s00464-025-12550-2.

Hands-on simulation training using human cadavers is an essential part of the training of medical specialists [1–3]. The high-fidelity aspect of human cadavers ensures that medical specialists receive realistic training early in their careers. The early training phase is where many mistakes are made, and cadaver training could help bridge the gap between early stage education and live surgery, reducing the risk of errors and patient harm [4, 5].

Despite the availability of other simulation techniques within laparoscopic surgery, such as virtual reality and dry lab training, cadaver training remains essential [6, 7]. Ideally, trainees should begin their education with dry lab training (box trainer, virtual reality simulator), followed by wet lab training (bio tissue or artificial tissue), before moving on to cadaver lab training, particularly in laparoscopic surgery [5, 6, 8]. Procedural skills and anatomical knowledge are ideally trained on human cadavers as these provide optimal simulation for the real situation, including tactile feedback, tissue and organ consistency, and realistic intra-abdominal color schemes [9, 10]. Furthermore, the human cadaver allows for the exploration of complex anatomical variations and pathologies that may not be encountered in live surgery. Realism is an important aspect of laparoscopic surgery training on human cadavers, enabling trainees to practice and develop their skills in conditions closely resembling those they will encounter in the operating room [3, 11, 12].

Fresh frozen and embalmed cadavers are the most commonly used types of human cadavers in laparoscopic training [13–16]. Fresh frozen cadavers are known for realistic tissue consistency, flexibility, and color, which closely mimics the properties of living tissue [9, 17]. However, fresh frozen cadavers have limitations, such as tissue damage during the freezing and thawing process, which can reduce the realism and face validity of the training experience. Another disadvantage is that the cadaver can only be used once or twice because of ongoing decomposition after defrosting, resulting in limited usage [14, 16, 17].

On the other hand, embalmed cadavers can be stored for longer periods without decomposition and can be used multiple times for surgical skills training [17, 18]. However, traditional embalmed cadavers can lead to discoloration and stiffening of the tissues, which are mainly caused by the combination of formaldehyde and phenol [17].

In other words, an embalming technique is needed that offers high realism and can be used multiple times for laparoscopic training. Fix4Life (F4L) is a novel embalming technique without phenol and with low formaldehyde concentration, resulting in flexible and pliable tissue without discoloration [3, 19]. Furthermore, the embalming allows long-term preservation and multiple uses, which are also relevant from an ethical and financial standpoint. The aim of this study was to assess the realism and face validity of the F4L embalmed human cadaver model for high-fidelity laparoscopic training.

## Methods

### Participants

Surgical residents from the Amsterdam University Medical Center (Amsterdam UMC) performed three types of laparoscopic procedures on F4L human cadavers during a hands-on laparoscopy course. These procedures were part of the Basic Laparoscopy Course and the Step by Step Inguinal Hernia Repair Course. The surgical residents consisted of a homogenous group of junior residents (PGY 1–3). Participants first completed a mandatory laparoscopic simulation course on laparoscopic box trainers with preset proficiency levels [5, 20]. After achieving the proficiency levels, the trainees were invited to the hands-on course at the Amsterdam Skills Centre [1]. Experts were defined as board-certified surgeons with > 5 years of laparoscopic practice.

### Ethical approval

Participation was voluntary, the data were anonymized, and there were no consequences. Written informed consent was acquired from all participants. The medical research ethics committee of the Amsterdam UMC was consulted regarding the study. The research proposal and protocol were assessed by the dedicated ethical committee, and approval was obtained on July 17, 2023.

### Laparoscopic procedures

**Fig. 1:**
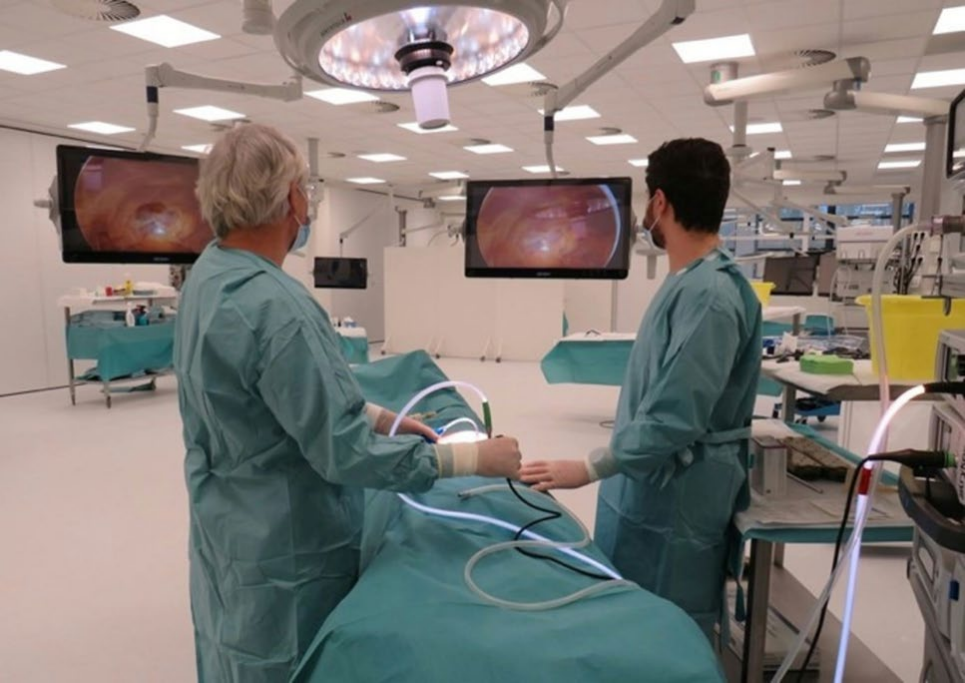
Laparoscopic training setup

Surgical residents performed three laparoscopic procedures: laparoscopic appendectomy, laparoscopic cholecystectomy, and totally extra-peritoneal (TEP) hernia repair (Fig. 1). The procedures were conducted under the supervision of five surgical faculty members following a theoretical step-by-step introduction and lecture.

All procedures were recorded, and the footage was saved on an external SSD connected to the laparoscopic tower (Stryker Corporation, Kalamazoo, Michigan, USA). The recordings were viewed and edited to ensure the removal of any identifiable information and extracorporeal images before being securely shared for assessment. The videos were assessed within an encrypted and secure online environment. Recording were automatically deleted after three months.

### Fix4Life setup

The Fix4Life specimens were prepared by anatomy technicians of the Department of Anatomy of the Amsterdam UMC and the Amsterdam Skills Centre. The embalming technique used a low concentration of formaldehyde and no phenol. Preparation costs were comparable to traditional embalming and due to the possibility of repeated use, Fix4Life embalmed specimens are more cost-effective in practice compared to Fresh Frozen specimens.

**Fig. 2:**
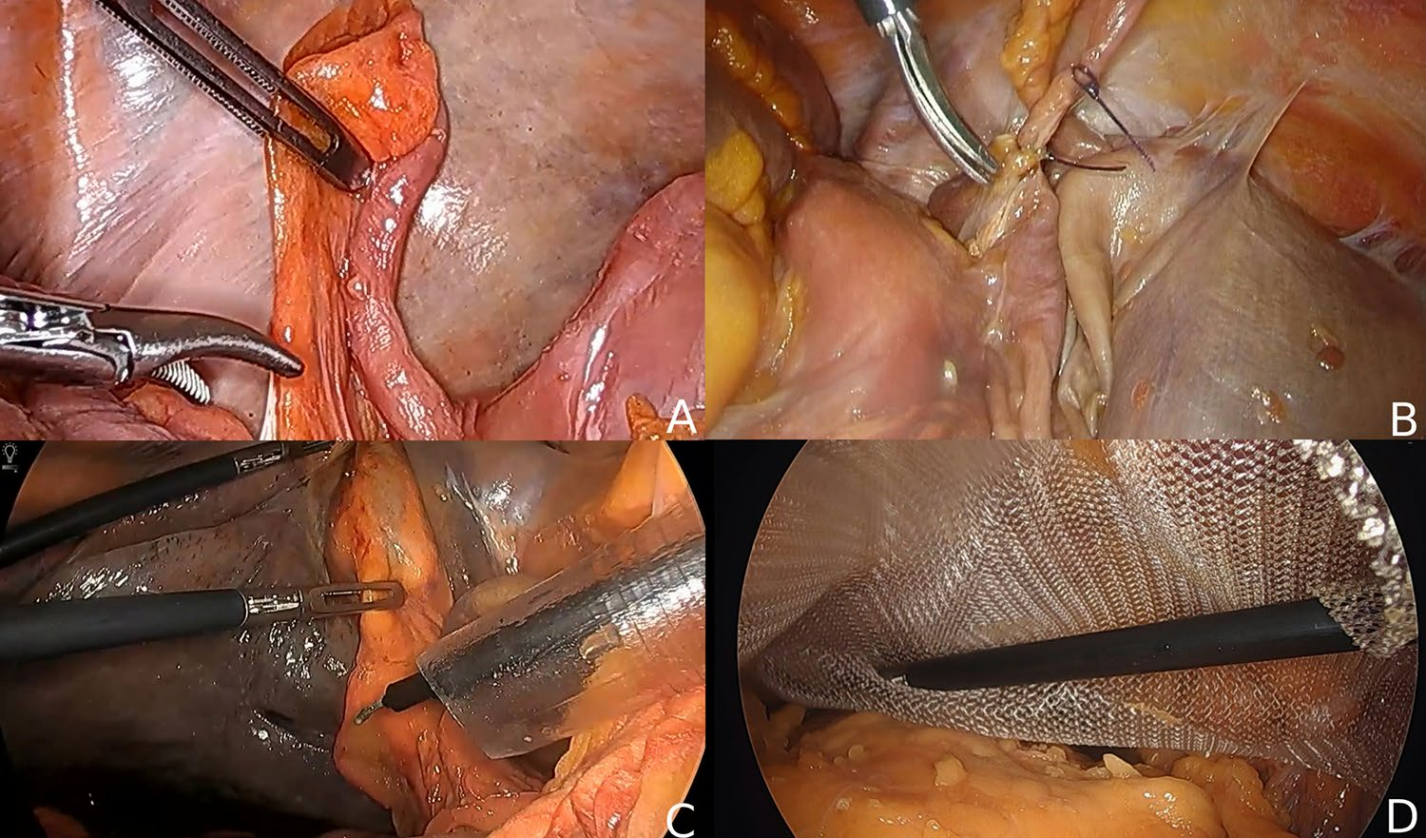
Laparoscopic images of the laparoscopic appendectomy (**A**, **B**), laparoscopic cholecystectomy (**C**), and TEP (**D**)

The Fix4Life specimen were draped as in the operating room (Fig. 2). The laparoscopic appendectomy, laparoscopic cholecystectomy, and TEP were performed using laparoscopic needle holders, laparoscopic curved dissecting forceps (Maryland), laparoscopic small and large fenestrated bowel graspers, and laparoscopic curved scissor (HiQ + instruments, Olympus corporation, Shinjuku, Tokyo, Japan). Additionally, Surgitie (Medtronic plc, Minneapolis, Minnesota, USA) was used during the laparoscopic appendectomy, and the Endo Clip (Medtronic plc, Minneapolis, Minnesota, USA) was used during the laparoscopic cholecystectomy.

### Face validation novices

After completing the laparoscopic training course, the residents filled out the face validation form (Table 1). The surgical faculty and the Department of Anatomy of Amsterdam UMC created the face validation form, which consisted of key aspects of laparoscopic surgery and realism. These aspects were assessed using a five-point Likert scale. The assessment form focused on visual realism, manipulation, technicalities such as insufflation and fulcrum effect, and an overall assessment of the Fix4Life training modality.

**Table 1:** Face validation form

Assessment	Very insufficient	Insufficient	Sufficient	Good	Very good
Laparoscopic view	1	2	3	4	5
Organ color	1	2	3	4	5
Tissue color	1	2	3	4	5
Organs reacts lifelike to manipulation	1	2	3	4	5
Tissue reacts lifelike to manipulation	1	2	3	4	5
Insufflation and pneumoperitoneum	1	2	3	4	5
Depth perception	1	2	3	4	5
Fulcrum effect					
The Fix4Life model is comparable to reality	1	2	3	4	5
The Fix4Life model is a valuable training modality to use prior to procedures on patients	1	2	3	4	5
Overall satisfaction	1	2	3	4	5

### Face validation experts

Following the laparoscopic course and an additional review of laparoscopic video recordings post-course, the surgical faculty also evaluated the realism of Fix4Life specimens as a training modality. All surgical faculty received the same instructions for filling out the form and reviewing the videos. The experts also signed a confidentiality statement, and access to the database was automatically removed after three months.

### Statistical tests

The Shapiro–Wilk test was used to test for normality, and the datapoints were significant on all points, resulting in non-normally distributed data. The Friedman test was used as non-parametric test to compare the three groups with a significance level of *p* < 0.05. The Wilcoxon Signed Rank test was used as post hoc test to determine the significant differences, and a Bonferroni adjustment was performed to account for multiple testing. The significance level of 0.05 was divided by the number of tests, which was 3, resulting in a significance level of *p* < 0.0167.

## Results

### Face validation by residents

In total, thirty surgical residents performed a laparoscopic appendectomy, laparoscopic cholecystectomy, and TEP, and filled out the face validation form (Table 2). The residents filled out a baseline demographics form, showing a homogenous group (47% male, 87% right dominant hand) with laparoscopic novices (Supplemental File, Table A1).

**Table 2:** Laparoscopic novice face validation

Assessment	Lap app (*n* = 30) Median (IQR)	Lap chol (*n* = 30) Mean (SD)	TEP (*n* = 30) Mean (SD)	Friedman test
Laparoscopic view	4.00 (1.00)	4.00 (1.00)	5.00 (1.00)	**0.024**
Organ color	4.00 (2.00)	4.00 (0.50)	4.00 (1.00)	0.285
Tissue color	4.00 (0.25)	4.00 (2.00)	5.00 (1.00)	**0.020**
Organs reacts lifelike to manipulation	5.00 (1.00)	4.00 (2.00)	4.50 (1.00)	**0.004**
Tissue reacts lifelike to manipulation	4.00 (1.00)	4.00 (1.25)	5.00 (1.00)	**0.012**
Insufflation and pneumoperitoneum	4.00 (1.00)	4.00 (1.00)	5.00 (1.00)	0.076
Depth perception	4.00 (1.00)	4.00 (1.00)	4.50 (1.00)	0.917
Fulcrum effect	4.00 (1.00)	4.00 (1.00)	4.00 (1.00)	0.887
The Fix4Life model is comparable to reality	4.00 (1.00)	4.00 (1.00)	5.00 (1.00)	0.113
The Fix4Life model is a valuable training modality to use prior to procedures on patients	4.00 (1.00)	4.00 (1.00)	4.50 (1.00)	0.835
Overall satisfaction	4.00 (1.00)	4.00 (1.00)	5.00 (1.00)	**0.009**

Bold indicates statistically significant at *p* < 0.05

The residents scored the laparoscopic appendectomy higher than 4, equaling to a rating of “Good” for all assessment points: laparoscopic view (4.30 Mean, SD 0.53), organ color (4.27 Mean, SD 0.74), tissue color (4.10 Mean, SD 0.67), lifelike manipulation of organs (4.27 Mean, SD 0.74), lifelike manipulation of tissue (4.23 Mean, SD 0.73), insufflation and pneumoperitoneum (4.20 Mean, SD 0.66), depth perception (4.43 Mean, SD 0.57), fulcrum effect (4.37 Mean, SD 0.56), comparability with reality (4.30 Mean, SD 0.70), value as a training modality (4.47 Mean, SD 0.51), and overall satisfaction (4.37 Mean, SD 0.49).

The laparoscopic cholecystectomy was also assessed as “Good” for all assessment points: laparoscopic view (4.13 Mean, SD 0.63), organ color (4.27 Mean, SD 0.58), tissue color (4.10 Mean, SD 0.71), lifelike manipulation of organs (3.93 Mean, SD 0.84), lifelike manipulation of tissue (4.07 Mean, SD 0.83), insufflation and pneumoperitoneum (4.10 Mean, SD 0.71), depth perception (4.37 Mean, SD 0.56), fulcrum effect (4.40 Mean, SD 0.56), comparability with reality (4.23 Mean, SD 0.63), value as a training modality (4.40 Mean, SD 0.56), and overall satisfaction (4.23 Mean, SD 0.57).

In the TEP, the organ color (4.43 Mean, SD 0.50), insufflation and pneumoperitoneum (4.47 Mean, SD 0.63), depth perception (4.40 Mean, SD 0.67), and the fulcrum effect (4.43 Mean, SD 0.50) were assessed as “Good”. The laparoscopic view (4.57 Mean, SD 0.50), tissue color (4.67 Mean, SD 0.48), lifelike manipulation of organs (4.50 Mean, SD 0.51), lifelike manipulation of tissue (4.60 Mean, SD 0.50), and overall satisfaction (4.67 Mean, SD 0.48) were assessed as “Very Good”.

Comparing the resident face validation results between the laparoscopic procedures demonstrated significant differences for the laparoscopic view (*p* = 0.024), tissue color (*p* = 0.020), lifelike manipulation of organs (*p* = 0.004), lifelike manipulation of tissue (*p* = 0.012), and overall satisfaction (*p* = 0.009). The TEP was assessed better than the laparoscopic cholecystectomy for laparoscopic view (*p* = 0.009), lifelike manipulation of organs (*p *= 0.002), lifelike manipulation of tissue (*p* = 0.005), and overall satisfaction (*p* = 0.005) (Supplemental File, Table A2). Furthermore, the tissue color in TEP was assessed better than both laparoscopic appendectomy (*p* = 0.011) and laparoscopic cholecystectomy (0.015). No significant differences were observed between the laparoscopic appendectomy and laparoscopic cholecystectomy.

### Face validation experts

In total, ten surgeons supervised the laparoscopic procedures and analyzed the videos (Table 3). The laparoscopic appendectomy was assessed as “Sufficient” for lifelike manipulation of organs (3.00 Median, IQR 1.00), “Good” for laparoscopic view (4.00 Median, IQR 2.00), organ color (4.00 Median, IQR 1.00), tissue color (3.50 Median, IQR 1.00), lifelike manipulation of tissue (4.00 Median, IQR 1.00), insufflation and pneumoperitoneum (4.00 Median, IQR 0.50), depth perception (4.00 Median, IQR 1.00), fulcrum effect (4.00 Median, IQR 1.00), comparability with reality (4.00 Median, IQR 1.25), and overall satisfaction (4.00 Median, IQR 1.25). The value as a training modality was rated as “Very Good” (5.00 Median, IQR 1.00).

**Table 3:** Laparoscopic expert face validation

Assessment	Lap app (*n* = 10) Mean (SD)	Lap chol (*n* = 10) Mean (SD)	TEP (*n* = 10) Mean (SD)	Friedman test
Laparoscopic view	4.00 (2.00)	4.00 (1.25)	5.00 (1.00)	0.142
Organ color (appendix, colon)	4.00 (1.00)	4.00 (1.25)	4.00 (1.25)	0.565
Tissue color	3.50 (1.00)	4.00 (1.00)	4.00 (1.25)	0.422
Organs reacts lifelike to manipulation (appendix, colon)	3.00 (1.00)	3.00 (1.25)	4.00 (0.50)	0.156
Tissue reacts lifelike to manipulation (mesoappendix dissection and ligatures)	4.00 (1.00)	3.00 (1.00)	4.00 (1.00)	**0.019**
Insufflation and pneumoperitoneum	4.00 (0.50)	4.00 (1.00)	4.00 (1.00)	0.074
Depth perception	4.00 (1.00)	4.50 (1.00)	4.00 (1.00)	0.549
Fulcrum effect (movement of instruments and trocar handling)	4.00 (1.00)	4.00 (1.00)	4.50 (1.00)	0.687
The Fix4Life model is comparable to reality	4.00 (1.25)	4.00 (1.00)	4.00 (1.25)	0.381
The Fix4Life model is a valuable training modality to use prior to procedures on patients	5.00 (1.00)	5.00 (1.00)	4.50 (1.00)	0.882
Overall satisfaction	4.00 (1.25)	4.00 (1.00)	4.00 (1.25)	0.565

Bold indicates statistically significant at *p* < 0.05

The laparoscopic cholecystectomy was assessed as “Sufficient” for lifelike manipulation of organs (3.00 Median, IQR 1.25) and lifelike manipulation of tissue (3.00 Median, IQR 1.00), and as “Good” for laparoscopic view (4.00 Median, IQR 1.25), organ color (4.00 Median, IQR 1.25), tissue color (4.00 Median, IQR 1.00), insufflation and pneumoperitoneum (4.00 Median, IQR 1.00), depth perception (4.50 Median, IQR 1.00), fulcrum effect (4.00 Median, IQR 1.00), comparability with reality (4.00 Median, IQR 1.00), and overall satisfaction (4.00 Median, IQR 1.00). The value as a training modality was again scored as “Very Good” (5.00 Median, IQR 1.00).

The surgeons assessed the TEP as “Good” for organ color (4.00 Median, IQR 1.25), tissue color (4.00 Median, IQR 1.25), lifelike manipulation of organs (4.00 Median, IQR 0.50), lifelike manipulation of tissue (4.00 Median, IQR 1.00), insufflation and pneumoperitoneum (4.00 Median, IQR 1.00), depth perception (4.00 Median, IQR 1.00), comparability with reality (4.00 Median, IQR 1.25), and overall satisfaction (4.00 Median, IQR 1.00). Furthermore, laparoscopic view (4.00 Median, IQR 1.00), fulcrum effect (4.50 Median, IQR 1.00), and value as a training modality (4.50 Median, IQR 1.00) were scored as “Very Good”.

Comparing the surgeons’ face validation results between the laparoscopic procedures, demonstrated significant differences for lifelike manipulation of tissue (*p* = 0.019), however when performing the sub-analysis with a Bonferroni adjustment for multiple testing, there were no significant differences (Supplemental File, Table A3).

### Comparison novices and experts

When comparing the novices’ and experts’ assessment of the laparoscopic appendectomy, there was a significant difference in lifelike manipulation of organs (*p* < 0.001) and comparability with reality (*p* = 0.046). In the laparoscopic cholecystectomy, the score for lifelike manipulation of tissue was significantly different (*p* = 0.036). Lastly, comparing the TEP scores, tissue color (*p* = 0.014) and lifelike manipulation of tissue (*p* = 0.024) were statistically significant Table 4. The differences were in higher scores by novices versus the lower scores by experts for the corresponding assessment and procedure.

**Table 4:** Man-Whitney U test, novice and expert comparison

Assessment	*p*-value appendectomy	*p*-value cholecystectomy	*p*-value TEP
Laparoscopic view	0.331	0.770	0.890
Organ color (appendix, colon)	0.315	0.198	0.083
Tissue color	0.177	0.286	**0.014**
Organs reacts lifelike to manipulation (appendix, colon)	** < 0.001**	0.315	0.062
Tissue reacts lifelike to manipulation (mesoappendix dissection and ligatures)	0.988	**0.036**	**0.024**
Insufflation and pneumoperitoneum	0.469	0.077	0.198
Depth perception	0.914	0.590	0.548
Fulcrum effect (movement of instruments and trocar handling)	0.939	0.612	0.770
The Fix4Life model is comparable to reality	**0.046**	0.866	0.102
The Fix4Life model is a valuable training modality to use prior to procedures on patients	0.450	0.414	1.000
Overall satisfaction	0.450	0.528	0.123

Bold indicates statistically significant at *p* < 0.05

## Discussion

This study demonstrated that Fix4Life is a viable and realistic training modality for laparoscopic appendectomy, laparoscopic cholecystectomy, and laparoscopic totally extraperitoneal (TEP) inguinal hernia repair. Both residents and surgeons rated Fix4Life as a valuable training modality with high satisfaction. The residents assessed the Fix4Life specimens for all procedures as “Good”, with the majority of the TEP assessment points as “Very Good”. The surgeons assessed the Fix4Life specimens for the majority of procedures as either “Good” or “Very Good”. However, the residents scored the procedures more positively. This was expected, as a certain learning curve of real procedures needs to be passed to determine the significant differences in procedural skills and intricacies between a cadaver simulation and a procedure in the operating room. Novices have fewer reference points from live surgery to detect subtle differences in tissue handling and color [10, 21]. Including experts was nevertheless essential, as their evaluations provide a benchmark grounded in extensive clinical experience.

Fix4life provides an alternative to on-the-job training and serves as a bridge between simulators and the operating room. Residents achieve required skill levels through digital and physical laparoscopic simulators before applying skills to Fix4Life specimens. The realism of Fix4Life also allows for realistic assessment of procedurals skills and feedback. Moreover, it provides an opportunity for exploration of anatomy and demonstration of pitfalls.

Laparoscopy training on box trainers, digital, or virtual trainers is also essential for fundamental laparoscopic skills development [22–25]. However, these training modalities focus more on developing technical laparoscopic skills such as hand–eye coordination, bimanual dexterity, depth perception, and instrument efficiency. Procedural skills are not trained to the same extent. Ideally, the transition to human cadaver training begins with achieving preset benchmarks for laparoscopic skills on both virtual and physical laparoscopic simulators. With acquired skills, they can then be applied during a real procedure on human cadavers.

Human cadaver training is essential in developing laparoscopic skills as it provides realistic tissue manipulation, instrument interactions, dissection, suturing, and procedure-specific steps [26]. Furthermore, it teaches important steps such as patient positioning, sterile draping, trocar placement, pneumoperitoneum creation, and the use of insufflation and diathermy [14, 21, 26, 27]. Another option would be animal labs; however, the anatomy is less realistic, with lower fidelity, and it also brings ethical considerations. Moreover, many studies have demonstrated the transferability of laparoscopic skills acquired with human cadaver training to the operating room [28–30].

The strength of Fix4Life lies in the fact that it offers a realistic training modality for laparoscopic training where a specimen can be reused multiple times, unlike Fresh Frozen [14]. For example, a laparoscopic TEP training can take place, and then later, a laparoscopic colectomy course or an orthopedic course. Additionally, it maintains the suppleness, elasticity, and color of the tissue compared to traditional embalming. This allows for realistic dissection and tissue manipulation in laparoscopic procedures. In a follow-up study the correlation between laparoscopic box trainer performance and laparoscopic procedures on Fix4Life will be compared.

## Conclusion

The Fix4Life embalmed method provides a realistic training modality for laparoscopic appendectomy, laparoscopic cholecystectomy, and TEP.

## Supplementary Information

Below is the link to the electronic supplementary material.

Supplementary file1 (DOCX 15 kb)

## References

[1] Yiasemidou M, Roberts D, Glassman D, Tomlinson J, Biyani S, Miskovic D (2017) A multispecialty evaluation of thiel cadavers for surgical training. World J Surg 41(5):1201–1207 28144746 10.1007/s00268-016-3868-4 PMC5394144

[2] Tjalma WA, Degueldre M, Van Herendael B, D’Herde K, Weyers S (2013) Postgraduate cadaver surgery: an educational course which aims at improving surgical skills. Facts Views Vision ObGyn 5(1):61 PMC3987353 24753929

[3] van Emden MW, Geurts JJ, Schober P, Schwarte LA (2020) Suitability and realism of the novel Fix for Life cadaver model for videolaryngoscopy and fibreoptic tracheoscopy in airway management training. BMC Anesthesiol 20(1):203 32799813 10.1186/s12871-020-01121-8 PMC7429731

[4] Jansen MM, Hazenberg CE, de Ruiter QM, van Hamersvelt RW, Bleys RL, van Herwaarden JA (2020) Feasibility of fresh frozen human cadavers as a research and training model for endovascular image guided interventions. PLoS ONE 15(11):e0242596 33254200 10.1371/journal.pone.0242596 PMC7704126

[5] Rahimi AM, Hardon SF, Uluç E, Bonjer HJ, Daams F (2023) Prediction of laparoscopic skills: objective learning curve analysis. Surg Endosc. 10.1007/s00464-022-09473-7 35927349 10.1007/s00464-022-09473-7 PMC9839814

[6] Holland JP, Waugh L, Horgan A, Paleri V, Deehan DJ (2011) Cadaveric hands-on training for surgical specialties: is this back to the future for surgical skills development? J Surg Educ 68(2):110–116 21338966 10.1016/j.jsurg.2010.10.002

[7] Takayesu JK, Peak D, Stearns D (2017) Cadaver-based training is superior to simulation training for cricothyrotomy and tube thoracostomy. Internal Emerg Med 12(1):99–102 27021389 10.1007/s11739-016-1439-1

[8] Sharma M, Macafee D, Horgan AF (2013) Basic laparoscopic skills training using fresh frozen cadaver: a randomized controlled trial. The Am J Surg 206(1):23–31 23623462 10.1016/j.amjsurg.2012.10.037

[9] Sharma M, Macafee D, Pranesh N, Horgan AF (2012) Construct validity of fresh frozen human cadaver as a training model in minimal access surgery. JSLS: J Soc Laparoendosc Surg 16(3):345 10.4293/108680812X13462882735818 PMC3535798 23318058

[10] Prasad Rai B, Tang B, Eisma R, Soames RW, Wen H, Nabi G (2012) A qualitative assessment of human cadavers embalmed by Thiel’s method used in laparoscopic training for renal resection. Anat sci educ 5(3):182–186 22362548 10.1002/ase.1267

[11] Rajasekhar SS, Kumar V, Raveendranath V, Kalayarasan R, Gnanasekaran S, Pottakkat B et al (2021) Advanced training in laparoscopic gastrointestinal surgical procedures using Genelyn®-embalmed human cadavers: a novel model. J Minim Access Surg 17(4):495–501 33605926 10.4103/jmas.JMAS_152_20 PMC8486066

[12] Katz R, Hoznek A, Antiphon P, Van Velthoven R, Delmas V, Abbou CC (2003) Cadaveric versus porcine models in urological laparoscopic training. Urol int 71(3):310–315 14512654 10.1159/000072684

[13] Hammer N, Löffler S, Bechmann I, Steinke H, Hädrich C, Feja C (2015) Comparison of modified Thiel embalming and ethanol-glycerin fixation in an anatomy environment: potentials and limitations of two complementary techniques. Anat Sci Educ 8(1):74–85 24706536 10.1002/ase.1450

[14] Hayashi S, Naito M, Kawata S, Qu N, Hatayama N, Hirai S et al (2016) History and future of human cadaver preservation for surgical training: from formalin to saturated salt solution method. Anat Sci Int. 10.1007/s12565-015-0299-5 26670696 10.1007/s12565-015-0299-5

[15] Ahmed K, Aydin A, Dasgupta P, Khan MS, McCabe JE (2015) A novel cadaveric simulation program in urology. J Surg Educ 72(4):556–565 25683152 10.1016/j.jsurg.2015.01.005

[16] Leblanc F, Champagne BJ, Augestad KM, Neary PC, Senagore AJ, Ellis CN et al (2010) A comparison of human cadaver and augmented reality simulator models for straight laparoscopic colorectal skills acquisition training. J Am Coll Surg 211(2):250–255 20670864 10.1016/j.jamcollsurg.2010.04.002

[17] James HK, Chapman AW, Pattison GTR, Griffin DR, Fisher JD (2019) Systematic review of the current status of cadaveric simulation for surgical training. Br J Surg 106(13):1726–1734 31573088 10.1002/bjs.11325 PMC6900127

[18] Giger U, Frésard I, Häfliger A, Bergmann M, Krähenbühl L (2008) Laparoscopic training on Thiel human cadavers: a model to teach advanced laparoscopic procedures. Surg endos 22(4):901–906 10.1007/s00464-007-9502-7 17704868

[19] Van Dam AJ et al (2022) Fix for life, a fresh look at body embalming and preservation. Anatomy Museum & Department of Anatomy and Embryology, Leiden University Medical Centre. Available from https://fixforlifeembalming.com/

[20] Rahimi AM, Hardon SF, Scholten SR, Bonjer HJ, Daams F (2023) Objective measurement of retention of laparoscopic skills: a prospective cohort study. Int J Sur 109(4):723–728 10.1097/JS9.0000000000000272 PMC10389389 37010141

[22] Sharma M, Macafee D, Horgan AF (2013) Basic laparoscopic skills training using fresh frozen cadaver: a randomized controlled trial. Am J Surg 206(1):23–31 23623462 10.1016/j.amjsurg.2012.10.037

[23] Meling TR, Meling TR (2021) The impact of surgical simulation on patient outcomes: a systematic review and meta-analysis. Neurosurg Rev 44(2):843–854 32399730 10.1007/s10143-020-01314-2 PMC8035110

[24] Harrington CM, Bresler R, Ryan D, Dicker P, Traynor O, Kavanagh DO (2018) The correlation between fundamental characteristics and first-time performance in laparoscopic tasks. Am J Surg 215(4):618–624 28624230 10.1016/j.amjsurg.2017.04.015

[25] Louridas M, Szasz P, De Montbrun S, Harris KA, Grantcharov TP (2016) Can we predict technical aptitude? A syste matic review. Ann Surg 263(4):673–691 26079898 10.1097/SLA.0000000000001283

[26] Munz Y, Kumar BD, Moorthy K, Bann S, Darzi A (2004) Laparoscopic virtual reality and box trainers: is one superior to the other? Surg Endosc 18(3):485–494 14752633 10.1007/s00464-003-9043-7

[27] Beger O, Karagül Mİ, Koc T, Kayan G, Cengiz A, Yılmaz ŞN et al (2020) Effects of different cadaver preservation methods on muscles and tendons: a morphometric, biomechanical and histological study. Anat Sci Int 95(2):174–189. 10.1007/s12565-019-00508-z 31691180 10.1007/s12565-019-00508-z

[29] Sharma M, Horgan A (2012) Comparison of fresh-frozen cadaver and high-fidelity virtual reality simulator as methods of laparoscopic training. World J Surg 36(8):1732–1737. 10.1007/s00268-012-1564-6 22484566 10.1007/s00268-012-1564-6

[30] Gilbody J, Prasthofer AW, Ho K, Costa ML (2011) The use and effectiveness of cadaveric workshops in higher surgical training: a systematic review. Ann R Coll Surg Engl 93(5):347 21943455 10.1308/147870811X582954 PMC3365449

[31] Hyltander A, Liljegren E, Rhodin PH, Lönroth H (2002) The transfer of basic skills learned in a laparoscopic simulator to the operating room. Surg Endosc 16(9):1324–1328 11988802 10.1007/s00464-001-9184-5

[32] Spiliotis AE, Spiliotis PM, Palios IM (2020) Transferability of simulation-based training in laparoscopic surgeries: a systematic review. Minim Invasive Surg. 10.1155/2020/5879485 32908700 10.1155/2020/5879485 PMC7468652

