# Supplementary material for: Assessing the realism and face validity of Fix For Life: an embalmed human cadaver model for high-fidelity laparoscopic training
Source: Surg Endosc. 2026 Jan 26;40(4):3132–9. doi: 10.1007/s00464-025-12550-2 (PMC13053444; doi:10.1007/s00464-025-12550-2)
Supplement: Supplementary file 1 — Supplementary file1 (DOCX 15 kb) [file 464_2025_12550_MOESM1_ESM.docx]

**Supplemental File**

**Table A1** Baseline demographics

| Demographics | Median (IQR)  (n=30) |
| --- | --- |
| Age | 31 (2) |
| Gender | 14 male (16 female) |
| Dominant hand | 26 right (4 left) |
| Number of lap app as primary surgeon | 1 (2) |
| Number of lap chol as primary surgeon | 0 (0) |
| Number of TEP as primary surgeon | 1 (2) |

| Assessment | Asymp. Sig. (2-tailed) |
| --- | --- |
| Laparoscopic view |  |
| Lap app and Lap chol | 0.251 |
| Lap app and TEP | 0.046 |
| Lap chol and TEP | **0.009** |
| Tissue color |  |
| Lap app and Lap chol | 0.808 |
| Lap app and TEP | **0.011** |
| Lap chol and TEP | **0.015** |
| Organs reacts lifelike to manipulation |  |
| Lap app and Lap chol | 0.068 |
| Lap app and TEP | 0.185 |
| Lap chol and TEP | **0.002** |
| Tissue reacts lifelike to manipulation |  |
| Lap app and Lap chol | 0.302 |
| Lap app and TEP | 0.033 |
| Lap chol and TEP | **0.005** |
| Overall satisfaction |  |
| Lap app and Lap chol | 0.334 |
| Lap app and TEP | 0.039 |
| Lap chol and TEP | **0.005** |

**Table A2** Novices Wilcoxon Signed Rank test with a Bonferroni adjustment defining significance as p<0.0167

| Assessment | Asymp. Sig. (2-tailed) |
| --- | --- |
| Tissue reacts lifelike to manipulation |  |
| Lap app and Lap chol | 0.024 |
| Lap app and TEP | 0.180 |
| Lap chol and TEP | 0.058 |

**Table A3** Experts Wilcoxon Signed Rank test with a Bonferroni adjustment defining significance as p<0.0167
